# Multiple Sulfur Acceptors Dock at IscS

**DOI:** 10.1371/journal.pbio.1000353

**Published:** 2010-04-13

**Authors:** Kira Heller

**Affiliations:** Freelance Science Writer, Oakland, California, United States of America

**Figure:**
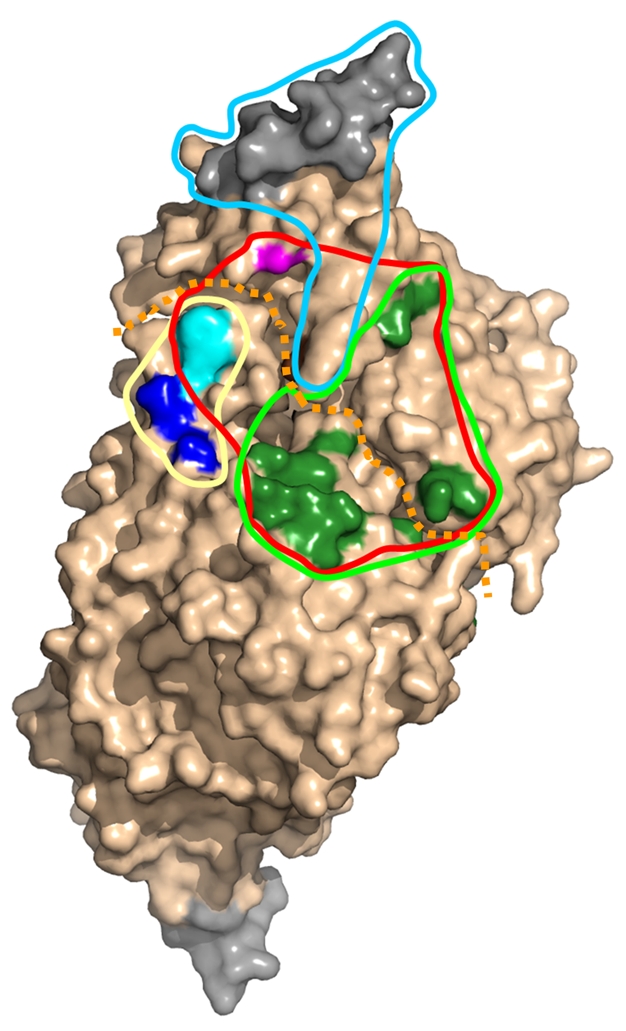
Crystal structures of a cysteine disulfurase bound to various sulfur-acceptor proteins reveal distinct binding footprints (solid lines) for the different partners, which all accept sulfur from the same active-site (magenta).

Like a busy dock, the enzyme IscS offers a temporary berth to multiple protein vessels where they can load up with their cargo of sulfur atoms. The sulfur is then incorporated into essential biological structures such as the iron–sulfur clusters that catalyze oxidation–reduction reactions in the electron transport chain. Although the structure of IscS has been known for some time, it was not clear how it recognizes and discriminates among the different incoming proteins. Do they dock at the same site on IscS, or do they have individual berths? Can they all moor at once, or do they have to wait their turn? In a new study in *PLoS Biology*, Rong Shi, Miroslaw Cygler, and colleagues investigated these questions in a series of crystallographic and biochemical experiments to map the surface of IscS and determine how it interacts with its various protein partners.

IscS is a highly conserved enzyme, present across phyla from bacteria to higher eukaryotes. In addition to assembly of iron–sulfur clusters in metalloproteins, IscS also participates in biological pathways such as the modification of tRNAs with sulfur (required for efficient codon recognition) and synthesis of sulfur-containing cofactors such as molybdenum cofactor (essential for the function of several enzymes). The researchers chose two proteins that accept sulfur from IscS: IscU, a scaffold for assembly of iron–sulfur clusters, and TusA, a sulfur acceptor that participates in a pathway that modifies several tRNAs by adding sulfur. They then crystallized each of them separately in a complex with IscS.

X-ray crystallography of the IscS–IscU and IscS–TusA complexes revealed that although the binding sites for IscU and TusA were both located in the area surrounding IscS's active site cysteine (Cys328), they didn't overlap. By analyzing the pattern of conserved surface amino acids, Shi et al. also realized there was a large, contiguous area of evolutionarily conserved amino acids surrounding Cys328 that was substantially larger than the binding sites of IscU and TusA.

To further explore the conserved area around Cys328 and determine if other IscS protein partners also docked there, the researchers created a series of IscS mutant proteins in which single amino acids distributed across the entire interacting surface were changed in ways that they predicted would affect the binding properties. They then measured how these mutations affected binding to IscU and TusA, and to other known IscS partners: ThiI, which modifies tRNA by adding sulfur to specific nucleotides and participates in synthesis of the vitamin thiamine; frataxin (CyaY in bacteria), which is probably an iron donor for iron–sulfur cluster assembly, and IscX, the function of which is unknown. They found multiple mutations that disrupted binding of IscS with its protein partners; the binding “footprints” of ThiI, frataxin/CyaY, and IscX overlapped substantially, whereas those of ThiI and TusA overlapped only partially.

Further binding experiments revealed that, unlike TusA and ThiI, IscU could bind to IscS at the same time as either frataxin/CyaY or IscX. In addition, TusA, IscU, and ThiI could not bind simultaneously to IscS, and IscU could displace TusA from IscS, suggesting that IscS has a higher affinity for IscU than for the other proteins. The researchers hypothesize that under conditions in which the supply of sulfur is limited, delivery to IscU, which is crucial to iron–sulfur cluster assembly, and therefore necessary for essential processes such as oxidative phosphorylation, would receive top priority.

In contrast to other cysteine desulfurases that interact with a single sulfur acceptor and have relatively short loops containing the active-site cysteine, Cygler's team discovered that the Cys328-containing loop in IscS was long and flexible. The length and flexibility of this loop could allow it to travel over relatively long distances to interact with IscS's binding partners.

By combining structural and biochemical experiments, this study has provided some hints about how IscS discriminates among its various binding partners; in the large, highly conserved docking surface surrounding Cys328, different proteins could take turns to moor in their respective berths. Once there, they would be approached by the flexible Cys328 “crane” to initiate transfer of the sulfur cargo. Finally, by favoring IscU under sulfur-deficient conditions, IscS can prioritize sulfur transfer to maintain essential biological processes such as energy production. The techniques that Shi et al. employed to probe the surface of IscS will likely be useful for investigating other macromolecules that interact with multiple proteins.

**Shi R, Proteau A, Villarroya M, Moukadiri I, Zhang L, et al. (2010) Structural Basis for Fe-S Cluster Assembly and tRNA Thiolation Mediated by IscS Protein-Protein Interactions. doi:10.1371/journal/pbio.1000354**

